# Psychological engagement with automated design improvement feedback: a multiple case study of ChatGPT in design education

**DOI:** 10.3389/fpsyg.2026.1729241

**Published:** 2026-02-16

**Authors:** Xin Wu, Yu Zeng, Zhirong Li, Xinyu Li, Siyu Chen, Longzhi Sun, Liangliang Zhu, Youngcheng Xie

**Affiliations:** 1 School of Architecture and Art, Central South University, Changsha, China; 2 School of Design, Raffles College of Higher Education, Singapore, Singapore; 3 School of Computer Science and Engineering, Central South University, Changsha, China; 4 College of Art and Design, Jiangsu University of Technology, Changzhou, China; 5 College of Computer Science and Technology, Zhejiang University, Hangzhou, China

**Keywords:** AI-assisted feedback systems, ChatGPT, design, education, psychological engagement

## Abstract

**Introduction:**

Understanding how students engage with AI-driven feedback remains understudied in educational psychology. With ChatGPT’s emergence as a generative artificial intelligence tool, automated design improvement feedback (ADIF) has expanded significantly. This exploratory study investigates differential engagement patterns with ChatGPT-based ADIF across performance levels, grounded in self-regulated learning theory.

**Methods:**

A mixed-method multiple case study examined 50 design students (25 high performers, 25 low performers) during a product design session. Data included behavioral observations of prompt strategies and query patterns, lag sequential analysis of cognitive transitions, and semi-structured interviews on emotional engagement.

**Results:**

High performers employed diverse prompt strategies with iterative refinement, exhibited cyclical metacognitive transitions, and characterized interactions as exploratory and collaborative. Low performers used basic prompts with limited iterations, demonstrated linear query-to-implementation progressions, and described structured guidance-seeking interactions.

**Discussion:**

The findings extend self-regulated learning theory to human-AI contexts, revealing how metacognitive capabilities shape behavioral, cognitive, and emotional engagement with AI feedback. Results demonstrate the need for scaffolding interventions to support lower-performing students in developing metacognitive strategies for effective AI interaction. This study contributes initial insights into performance-based variations in human-AI collaboration within educational contexts.

## 1 Introduction

Psychological engagement, as a core construct in educational psychology, encompasses behavioral manifestations, cognitive processes, and emotional responses that collectively demonstrate strong associations with learning outcomes (Heilporn et al., 2021). In interactive learning environments, effective feedback mechanisms have been established as key factors in enhancing student engagement. This is particularly evident in design education contexts, where feedback promotes cognitive development and reflective capabilities while facilitating iterative design thinking processes (Momose et al., 2025; Wang et al., 2025). In recent years, research focus has shifted from examining students’ attitudes toward feedback to understanding their feedback processing and application mechanisms (Van der Kleij and Lipnevich, 2021). Understanding the psychological mechanisms underlying student engagement with feedback represents a critical priority for advancing both educational psychology theory and pedagogical practice.

The emergence of ChatGPT as a generative artificial intelligence (GAI) tool has fundamentally transformed feedback delivery mechanisms and psychological dynamics in design education (Quan et al., 2023). Unlike traditional automated design improvement feedback (ADIF) systems, ChatGPT demonstrates advanced capabilities in providing contextualized, interactive feedback through natural language dialogue, creating qualitatively different interaction patterns compared to earlier feedback technologies (Lee and Chung, 2024). However, this technological shift introduces new psychological challenges for student engagement that require systematic investigation.

Despite growing interest in AI-assisted education, significant gaps remain in understanding the psychological mechanisms of student engagement with ChatGPT feedback in design contexts. First, existing research has predominantly employed large-scale quantitative methods that capture behavioral patterns but overlook the nuanced cognitive and emotional processes underlying human-AI interaction in creative domains (Lo et al., 2024). Second, while performance-level differences are well-documented in traditional feedback settings, how individual differences in design capabilities systematically shape engagement strategies with conversational AI systems remains unexplored. Third, the unique characteristics of design education, combining subjective aesthetic judgment, ill-structured problems, and creative uncertainty, create distinct challenges for AI feedback that have not been systematically examined from an educational psychology perspective. Most critically, current research lacks a theoretically grounded understanding of how students with different performance levels navigate the complex psychological landscape of AI-mediated design feedback, particularly regarding metacognitive regulation patterns and emotional negotiation of AI’s inherent limitations in subjective judgment.

The Chinese higher education context warrants particular attention in this investigation. Existing research on AI-assisted feedback has predominantly emerged from Western educational settings. However, Chinese design education exhibits distinctive characteristics that may shape engagement patterns in ways that existing frameworks do not fully capture. These characteristics include emphasis on formative assessment, technology-enhanced pedagogies, and unique cultural dynamics in teacher-student relationships. Recent studies have begun examining Chinese university students’ ChatGPT adoption and perceptions (Liu and Zhang, 2024; Shahzad et al., 2024). Yet empirical research specifically examining how Chinese design students psychologically engage with AI-generated feedback remains limited.

This study addresses these critical gaps through a mixed-method multiple case study investigating how design students with different performance levels engage with ChatGPT-generated automated design improvement feedback (ADIF). The investigation is both timely and significant for several reasons. Theoretically, it extends self-regulated learning theory to human-AI interaction contexts, revealing how individual differences in metacognitive capabilities systematically shape behavioral strategies, cognitive processing patterns, and emotional responses when engaging with conversational AI systems. Methodologically, it demonstrates the value of in-depth case study approaches for uncovering psychological mechanisms that large-scale studies may overlook, particularly in emerging technological contexts where interaction patterns are still evolving. Practically, it provides evidence-based insights for designing psychologically-informed scaffolding strategies when deploying AI feedback systems, with particular attention to supporting lower-performing students in developing metacognitive and strategic capabilities essential for effective AI interaction. These contributions are particularly urgent given the rapid adoption of ChatGPT in educational settings without sufficient understanding of its psychological implications across diverse student populations. Specifically, this study addresses three research questions (RQs):

RQ1: How do design students with different performance levels behaviorally engage with ChatGPT feedback?

RQ2: What cognitive processes do they employ when processing AI-generated feedback?

RQ3: How do emotional responses to AI feedback vary across performance levels?

## 2 Literature review

### 2.1 The development and potential of ADIF in design practice environments

Design education environments have witnessed a significant transformation through the integration of automated design improvement feedback (ADIF) systems. These systems, defined as computerized tools providing design improvement suggestions, have evolved to address the growing needs for timely, consistent, and personalized feedback in interactive design learning contexts within higher education (Luo et al., 2025).

Large Language Models (LLMs) are artificial intelligence systems trained on massive text corpora through deep learning, enabling them to understand context, generate coherent text, and engage in multi-turn conversations (Bhattacharya et al., 2024). The emergence of ChatGPT as a large language model (LLM)-based system marks a qualitative shift in ADIF systems’ capabilities. Unlike traditional rule-based or machine learning systems that primarily evaluate technical aspects like layout and typography, ChatGPT demonstrates unprecedented capabilities in providing contextualized, interactive feedback across multiple design dimensions. Recent empirical studies highlight ChatGPT’s distinct advantages in supporting design education, particularly in fostering concept development, creative inspiration, and interdisciplinary thinking (Lee and Chung, 2024). The conversational nature of ChatGPT enables iterative feedback refinement, allowing students to engage in dialogue-like interactions that more closely mirror traditional designer-mentor relationships (Filippi, 2023).

However, understanding ChatGPT’s role in design education requires acknowledging fundamental epistemological differences between AI-generated and human feedback. Human feedback in design is grounded in embodied experience, tacit knowledge, and aesthetic sensibility developed through years of practice and cultural immersion (Cross, 2004). Expert design mentors draw upon intuitive judgment that integrates subjective experience with analytical reasoning in ways that resist algorithmic replication. AI systems, by contrast, generate feedback through statistical pattern recognition in training data, operating without phenomenological experience of aesthetic qualities or authentic cultural understanding (Bender et al., 2021). While ChatGPT can reference design principles and analyze precedents, it cannot authentically engage with the subjective dimensions that designers navigate, such as the felt sense of visual harmony or the cultural weight of symbolic choices. This epistemological distinction fundamentally shapes how AI feedback should be understood and utilized in design education, creating unique challenges for student engagement that warrant systematic investigation.

Additionally, ChatGPT’s implementation in design education contexts presents unique challenges that warrant careful investigation. Research indicates that the effectiveness of ChatGPT-generated feedback heavily depends on students’ ability to articulate design concepts and construct appropriate prompts (Jukiewicz, 2024). Moreover, the system may generate what researchers term “creative hallucinations” — suggestions that appear innovative but lack practical feasibility (Bhaskar and Gupta, 2024). These challenges are particularly pronounced in design education environments, where problems are typically ill-structured, and solutions often require multidimensional creative thinking.

The complex nature of human-ChatGPT interactions in design education necessitates research approaches that can capture nuanced interaction patterns and learning mechanisms. While large-scale studies provide valuable statistical insights, they often fail to reveal the underlying processes of how students engage with AI feedback (Lo et al., 2024). Case study methodology offers particular value in this context, enabling researchers to conduct in-depth investigations of how students navigate these interactions in naturalistic settings (Gerring, 2004). This approach is especially crucial given that the relationship between students’ design capabilities and their ability to effectively utilize ChatGPT remains poorly understood.

The rapid evolution of AI capabilities in design education environments also raises critical questions about how different students adapt to and benefit from these tools. Research suggests that effective use of ChatGPT requires not only AI literacy but also the capacity to critically evaluate and integrate AI suggestions with inherent creativity (Adeshola and Adepoju, 2024). Understanding these adaptation processes requires close examination of individual cases to reveal how students of varying abilities develop strategies for utilizing AI feedback in their design education.

### 2.2 The subjectivity challenge in AI-mediated design feedback

Design feedback inherently involves subjective interpretation across multiple dimensions, including aesthetic preference, cultural resonance, contextual appropriateness, and emotional impact (Christensen and Ball, 2016). Research in design cognition demonstrates that expert designers develop what Cross (2004) terms “designerly ways of knowing,” a form of tacit knowledge that integrates analytical reasoning with intuitive judgment accumulated through extensive practice. This subjective dimension is not peripheral but central to design quality. As Schön (2017) articulated, design practice involves “reflection-in-action” where practitioners engage in continuous dialogue with materials and situations, making judgments that resist explicit formalization. In educational contexts, feedback on these practices demands that mentors recognize nuanced qualities, such as whether a color palette evokes the intended emotional response or whether a form language aligns with cultural expectations, judgments that embody expertise rather than rule-based evaluation.

Conversational AI systems like ChatGPT face fundamental limitations in addressing design’s subjective dimensions. Despite impressive natural language capabilities, these systems operate through pattern matching in training data without phenomenological experience of aesthetic qualities (Bender et al., 2021). ChatGPT can discuss color theory principles but cannot experience how a particular blue feels calming or energizing. It can reference cultural symbolism but lacks the lived cultural experience that shapes authentic interpretation. Recent research on AI creativity highlights this gap, noting that while AI can generate novel combinations, it cannot evaluate aesthetic quality through subjective experience (Magni et al., 2024). In design feedback contexts, this limitation means AI systems may offer technically sound suggestions while missing subjective nuances that human mentors would immediately recognize. For instance, ChatGPT might recommend increasing contrast for readability without recognizing that subtle, low-contrast aesthetics are intentional choices conveying sophistication in luxury brand design.

The implications for student psychological engagement are significant yet underexplored. Students must navigate a complex evaluative landscape where AI feedback may be technically proficient but contextually or aesthetically inappropriate. This navigation requires students to develop critical evaluation skills beyond traditional feedback literacy, specifically the capacity to identify when AI suggestions align with subjective design intentions and when they miss crucial contextual factors (Suriano et al., 2025). The challenge is compounded by what researchers term “automation bias,” the tendency to overvalue algorithmically generated suggestions even when human judgment would be more appropriate (Glikson and Woolley, 2020). For novice designers still developing their own aesthetic judgment, distinguishing between technically correct but aesthetically inappropriate AI suggestions and genuinely valuable feedback presents a substantial cognitive and emotional challenge. How students across different performance levels psychologically negotiate this challenge, particularly regarding their emotional trust in AI feedback and cognitive strategies for evaluating subjective dimensions, remains a critical gap in current research. Understanding these processes is essential for supporting students in developing both AI literacy and design judgment simultaneously.

### 2.3 Student engagement with AI feedback: current knowledge and critical gaps

Student engagement, encompassing behavioral, cognitive, and emotional dimensions, is fundamental to designing education environments and learning outcomes (Reeve et al., 2020). With the integration of AI-enhanced feedback systems like ChatGPT, understanding how students engage with these tools has become increasingly crucial for design education contexts.

Current research has revealed distinct patterns in how students engage with AI-enhanced design feedback across different dimensions. From a behavioral perspective, studies suggest that engagement patterns are closely tied to students’ design capabilities and learning strategies. High-performing students tend to use AI feedback as a supplementary tool for design refinement, while lower-performing students often show greater dependency on AI suggestions (Pan, 2023). This behavioral distinction manifests in both feedback-seeking strategies and how students translate AI suggestions into design modifications. Recent studies on ChatGPT use in educational settings indicate that effective behavioral engagement requires sophisticated prompt engineering skills and iterative refinement strategies (Yan, 2025). Students who construct detailed, context-rich prompts receive more relevant feedback, creating engagement patterns that may differ substantially from traditional feedback interactions (Bhattacharya et al., 2024). However, current research predominantly examines single-session interactions or aggregated behavioral metrics, leaving critical gaps in understanding how behavioral engagement patterns evolve within complete design tasks and how these patterns differ systematically between high and low performers in design-specific contexts. The behavioral mechanisms through which students learn to effectively interact with conversational AI during authentic design work remain underspecified.

In terms of cognitive engagement, research has highlighted the critical role of students’ metacognitive abilities in processing AI feedback. A recent systematic review has identified feedback clarity as a key factor influencing students’ cognitive engagement with AI systems (Shi and Aryadoust, 2024). Furthermore, it was found that students’ perception of AI system reliability significantly impacts their depth of cognitive processing (Melker et al., 2025; Ng et al., 2024). These findings suggest that effective cognitive engagement with AI feedback requires not only understanding the feedback content but also the ability to evaluate and selectively implement AI suggestions. Emerging research on ChatGPT in educational contexts reveals that effective cognitive engagement requires students to employ critical evaluation strategies, distinguishing between technically sound but contextually inappropriate suggestions and genuinely valuable feedback (Suriano et al., 2025). This evaluative demand is particularly pronounced in design domains where multiple valid solutions exist and subjective judgment plays a central role. However, critical gaps persist in understanding the cognitive processes underlying student behaviors. Current research has not examined how students transition between different cognitive states, such as query formulation, feedback processing, and implementation decision-making, during AI-mediated design tasks. More importantly, whether and how these cognitive processing patterns differ between high and low performers remains unexplored, limiting our understanding of the cognitive mechanisms that distinguish effective from ineffective AI feedback engagement.

Regarding emotional engagement, recent studies have revealed complex patterns in students’ affective responses to AI feedback systems. While students generally show positive initial reactions to AI-assisted design support, their substantial cognitive engagement with the feedback often remains insufficient (Momose et al., 2025). Research indicates that students’ emotional attitudes toward AI feedback evolve with experience, transitioning from initial novelty to more rational assessments (Glikson and Woolley, 2020). Recent studies document that students often maintain ambivalent attitudes, appreciating AI guidance while expressing concerns about potential impacts on their creative development and authentic learning (Magni et al., 2024). This ambivalence may be particularly pronounced in design education, where personal creative identity and ownership of ideas are central to student self-concept (Rezai et al., 2024). Additionally, the “black box” nature of AI systems affects emotional trust, with students reporting uncertainty about how to evaluate the reliability of algorithmically generated suggestions (Lo et al., 2024). However, significant gaps remain in understanding how emotional responses vary across student performance levels and how these emotional patterns interact with behavioral and cognitive engagement. The emotional mechanisms through which students negotiate trust, anxiety, and creative confidence when using AI feedback in design tasks require systematic investigation.

Nevertheless, several significant gaps exist in current research. First, existing studies have predominantly focused on quantitative measures, potentially missing the nuanced ways students interact with AI systems in authentic design contexts (Yan and Zhang, 2024). Second, most research has examined engagement in relatively structured tasks, leaving the complex dynamics of AI-supported design processes underexplored. Third, while previous studies have investigated individual engagement dimensions separately, there is limited understanding of how these dimensions interact within the context of AI-enhanced design education environments (Lo et al., 2024).

These research gaps are particularly significant given the unique characteristics of design education environments, where problems are typically ill-structured, and solutions require iterative refinement. The introduction of conversational AI systems like ChatGPT further complicates this landscape by creating more dynamic and interactive feedback environments. Understanding how students navigate these complex interactions requires research approaches that can capture the multifaceted nature of engagement while accounting for individual differences in design capabilities and AI literacy.

### 2.4 Performance-level differences: an underexplored moderating factor in AI feedback engagement

Individual differences in performance level fundamentally shape how students engage with feedback, a relationship well-established in self-regulated learning (SRL) theory (Zimmerman, 2013). SRL framework posits that high and low performers differ systematically in metacognitive monitoring, strategic planning, and emotional regulation capabilities. High performers typically demonstrate superior metacognitive awareness, enabling them to set appropriate goals, monitor progress effectively, and adjust strategies when needed (Broadbent and Poon, 2015). They engage in what Zimmerman terms “forethought,” proactively planning their approach to learning tasks, and “self-reflection,” critically evaluating outcomes to inform future actions. In contrast, low performers often exhibit weaker metacognitive skills, struggling with goal setting, progress monitoring, and strategic adjustment (Fritzsche et al., 2018). These differences manifest across all three phases of SRL, namely forethought, performance, and self-reflection, affecting how students seek, process, and implement feedback. In traditional design education contexts, these performance-level differences are evident in feedback-seeking behaviors, with high performers asking more specific questions and low performers making more general requests (Pi et al., 2024). However, the introduction of AI feedback systems introduces qualitatively new cognitive demands that may interact with these existing differences in unexpected ways.

ChatGPT-mediated feedback in design education introduces cognitive demands distinct from traditional feedback mechanisms. Students must construct effective prompts that clearly communicate design intentions, context, and specific feedback needs, a skill requiring metacognitive awareness of both their design thinking and the AI system’s requirements (Yan, 2025). They must engage in iterative refinement, recognizing when initial AI responses are insufficient and formulating follow-up queries that progressively narrow toward useful feedback. Additionally, students must critically evaluate AI-generated suggestions, determining their relevance, feasibility, and alignment with subjective design goals, a task requiring both design expertise and understanding of AI limitations (Adeshola and Adepoju, 2024). These demands may particularly challenge low performers who already struggle with metacognitive regulation. Recent research suggests that effective ChatGPT use correlates with metacognitive skills, with students who can articulate their knowledge gaps and learning needs obtaining more valuable feedback (Koltovskaia et al., 2024). This relationship implies that AI feedback systems, rather than equalizing educational opportunities, might inadvertently amplify existing performance gaps if lower-performing students lack the metacognitive foundations necessary for effective AI interaction.

Despite the theoretical importance of performance-level differences, current research on AI feedback in design education has largely overlooked this moderating factor. Most studies either examine aggregated patterns across mixed-ability samples or focus exclusively on advanced students, leaving critical questions unanswered. How do behavioral strategies for seeking and implementing ChatGPT feedback differ between high and low performers in design tasks? Do cognitive processing patterns, such as transitions between query formulation, feedback evaluation, and implementation, vary systematically by performance level? How do emotional responses to AI feedback, including trust, anxiety, and creative confidence, differ across the performance spectrum? These gaps are particularly concerning given the rapid adoption of AI feedback tools in design education. Without understanding how these tools affect diverse student populations, we risk implementing interventions that benefit already high-performing students while providing insufficient support for those who most need assistance. Furthermore, the interaction between performance level and the unique challenges of AI-mediated design feedback, such as navigating subjective judgment and prompt engineering demands, remains largely unexplored. This gap is particularly evident within the Chinese educational context, where empirical investigations of student engagement with conversational AI in design education remain scarce despite growing scholarly attention to ChatGPT adoption among Chinese university students more broadly. Addressing this gap requires an in-depth investigation that can capture the nuanced ways different students psychologically engage with AI feedback systems across behavioral, cognitive, and emotional dimensions.

### 2.5 Theoretical framework

The preceding review reveals three critical and interconnected gaps in current understanding of student engagement with AI-driven feedback in design education. First, existing research has predominantly employed large-scale quantitative approaches that capture behavioral patterns and attitudinal measures but overlook the nuanced psychological processes, particularly cognitive state transitions and emotional negotiations, that characterize moment-to-moment engagement during authentic design tasks. This methodological limitation leaves the psychological mechanisms underlying effective versus ineffective AI feedback engagement largely unspecified. Second, despite well-established performance-level differences in traditional feedback contexts, how these differences manifest in AI-mediated design feedback remains unexplored. The unique cognitive demands of prompt engineering, iterative refinement, and critical evaluation of algorithmically generated content may interact with existing metacognitive capabilities in ways that current research has not examined. Third, the distinctive characteristics of design education, particularly the centrality of subjective aesthetic judgment and the epistemological limitations of AI in evaluating subjective dimensions, create challenges for student engagement that have not been systematically investigated from an educational psychology perspective. These gaps collectively limit our ability to understand how students psychologically navigate AI-mediated design feedback and how to support diverse learners in developing effective engagement strategies.

To address these gaps, this study integrates self-regulated learning theory with Ellis (2010) feedback typology to develop a comprehensive model for understanding student engagement with ChatGPT in design education. Self-regulated learning theory is particularly appropriate because it accounts for individual differences in metacognitive capabilities while providing a process-oriented framework that aligns with the iterative nature of both design work and ChatGPT interaction (Broadbent and Poon, 2015). The feedback typology complements this framework by offering specific mechanisms for analyzing how students process different types of AI-generated suggestions in design contexts. This integrated approach enables examination of how students’ self-regulatory capacities shape their behavioral, cognitive, and emotional engagement with ChatGPT feedback throughout the cyclical phases of forethought, performance, and self-reflection.

Self-regulated learning theory provides the overarching framework for understanding how students manage their learning processes through three cyclical phases, namely forethought, performance and self-reflection (Zimmerman, 2013). This theoretical lens aligns with the iterative nature of design work and accounts for individual differences in self-regulation capabilities that distinguish high from low performers (Broadbent and Poon, 2015). Within this SRL framework, Ellis’s (2010) feedback typology offers specific mechanisms for analyzing how students process different types of AI-generated suggestions in design contexts.

The proposed model conceptualizes engagement across three interconnected dimensions that map onto the SRL phases. Behavioral engagement, emerging primarily from the forethought phase, encompasses not only traditional feedback processing actions but also the unique aspect of composing prompts to obtain feedback from ChatGPT. Unlike traditional ADIF systems, the quality and relevance of ChatGPT’s feedback heavily depends on students’ ability to construct effective prompts through iterative and incremental interactions (Bhattacharya et al., 2024). The dimension reflects students’ strategic planning and goal-setting capabilities in their approach to AI interaction.

Cognitive engagement, corresponding to the performance phase of SRL, is conceptualized as students’ utilization of cognitive and metacognitive strategies when processing ChatGPT feedback. The dimension builds upon previous research but extends it to account for the conversational nature of ChatGPT interactions (Yan and Zhang, 2024). The model emphasizes how students navigate between different cognitive states during their dialogue with ChatGPT, from understanding feedback to implementing design changes within a single design task. High-performing students are expected to demonstrate superior metacognitive monitoring during this phase, enabling more effective evaluation of AI feedback quality and relevance (Mertens et al., 2022).

Emotional engagement, crystallizing during the self-reflection phase, represents students’ affective responses and attitudes toward ChatGPT-generated feedback. The dimension is crucial in design education environments, where creative expression and personal investment in design solutions play significant roles. The model accounts for how students’ emotional responses develop during their interactions with ChatGPT, from initial reactions to trust and reliance patterns that emerge throughout the design process. These emotional responses subsequently influence future cycles of engagement, creating feedback loops that shape long-term interaction patterns.

**Figure 1:**
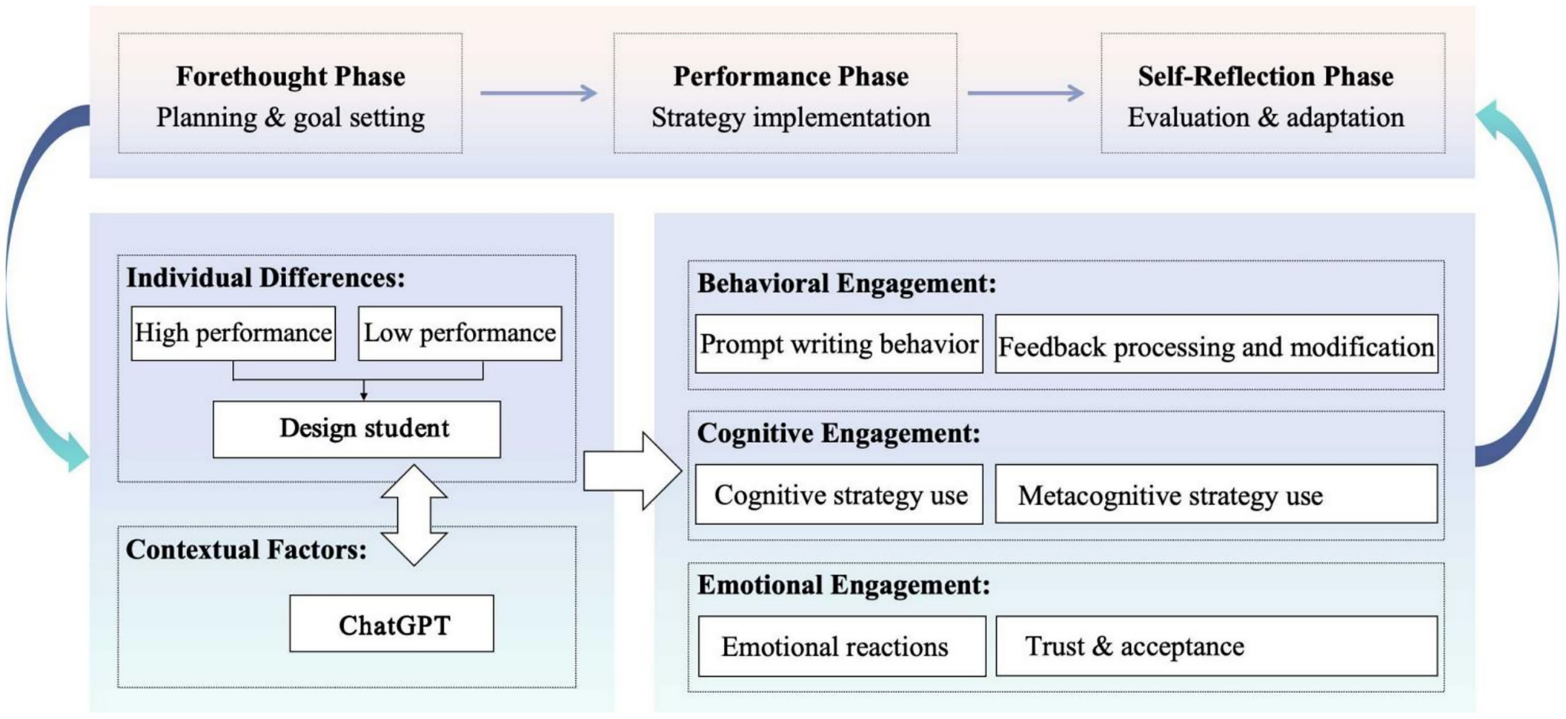
ADIF systems model of ChatGPT engagement.

The interrelationships between these dimensions form a dynamic system where each dimension influences the others across the learning cycle. Students’ emotional responses to ChatGPT may influence their behavioral patterns in constructing prompts, which in turn affects their cognitive processing of the received feedback. The cyclical interaction pattern, moderated by students’ performance levels, distinguishes the current model from previous frameworks that often treated these dimensions as separate entities. Figure 1 illustrates these relationships and the central role of performance level in shaping engagement patterns.

Given these theoretical foundations and identified gaps, this study employs a multiple case study approach to provide an in-depth examination of how design students with different performance levels engage with ChatGPT-generated feedback. Case study methodology is particularly appropriate for this exploratory investigation as it enables detailed observation of complex psychological processes in naturalistic settings, capturing nuances that large-scale studies may overlook (Gerring, 2004). By focusing on behavioral strategies, cognitive processing patterns, and emotional responses across performance levels, this investigation addresses the critical need for process-oriented understanding of human-AI interaction in design education contexts. The investigation addresses three specific research questions outlined in the Introduction. The investigation addresses three specific research questions that directly correspond to the identified gaps. RQ1 examines behavioral engagement patterns to address the gap in understanding how prompt construction and feedback implementation strategies differ across performance levels. RQ2 investigates cognitive processing patterns to reveal the metacognitive mechanisms underlying effective versus ineffective AI interaction. RQ3 explores emotional responses to understand how students negotiate trust and creative anxiety when engaging with AI’s subjective limitations. By focusing on these behavioral, cognitive, and emotional dimensions across performance levels, this investigation provides the process-oriented understanding of human-AI interaction in design education that current research lacks.

### 2.6 Research hypotheses

Based on the theoretical foundation established above and prior research on performance differences in technology-enhanced learning, we propose the following research hypotheses:

*H1* (Behavioral engagement): Higher-performing students are expected to demonstrate more sophisticated feedback-seeking strategies, characterized by diverse prompt construction types and iterative query refinement, compared to lower-performing students.

*H2* (Cognitive engagement): Higher-performing students are expected to exhibit cyclical cognitive processing patterns reflecting metacognitive monitoring and regulation, while lower-performing students are expected to show more linear progression patterns.

*H3* (Emotional engagement): Performance levels are expected to be associated with differential emotional responses to AI feedback, with higher performers likely perceiving AI interaction as collaborative exploration and lower performers as structured guidance-seeking.

## 3 Methods

### 3.1 Participants

This study involved human participants. The protocol was approved by the Ethical Committee of the participating university. The study was conducted at the College of Art and Design, Jiangsu University of Technology, China. The participants were third-year undergraduate students majoring in visual communication design. The core curriculum of this major employs formative assessment and technology-enhanced feedback teaching methods. The participants had completed an average of eight major courses using these teaching methods over the past four semesters, thus gaining substantial experience in design-oriented assessment practices (Table 1).

**Table 1:** Participant characteristics and performance classification criteria.

Characteristic	High performers	Low performers
Gender distribution	Male: 26, Female: 24	
Academic level	Third-year undergraduate	
Major	Visual communication design	
Prior experience with design feedback	8 courses over 4 semesters	
Total sample	*n* = 25	*n* = 25
Design course GPA (2-year average)	≥3.5/4.0	≤2.8/4.0
Technical proficiency score	≥85/100	≤70/100
Composite performance score	≥80	≤65
Inter-rater reliability (Cohen’s Kappa)	κ = 0.88	

The selection of participants employed a stratified purposive sampling method. The selection of participants employed a comprehensive academic performance evaluation framework. Student performance was assessed through two main components: (i) Academic achievement (50%) - including design course Grade Point Average (GPA) over the past 2 years (≥3.5/4.0 for high performers, ≤2.8/4.0 for low performers); and (ii) Technical proficiency (50%) - assessed through standardized tests in Adobe Creative Suite (25%) and UI/UX design tools (25%), with clear performance benchmarks (≥85/100 for high performers, ≤70/100 for low performers). After weighted calculation, participants scoring ≥80 were classified as high performers, while those scoring ≤65 were classified as low performers. Two evaluators conducted all assessments, achieving an inter-rater reliability coefficient of 0.88 (Cohen’s Kappa).

Of the 78 volunteers, the research team categorized students into high performers and low performers based on these dimensions. Following a preliminary screening process, 50 students were selected as research participants, with 25 high performers and 25 low performers. The decision to select 50 participants was guided by three considerations: (i) methodological appropriateness - as an in-depth exploratory study focusing on complex interaction patterns, this sample size allows for thorough data collection and analysis while maintaining statistical power for meaningful comparisons between performance groups; (ii) data richness - each participant generated comprehensive observation data, interview transcripts, and reflection journals during the single design session, providing sufficient depth for meaningful analysis; and (iii) theoretical sampling - the selected participants represented distinct profiles in terms of performance level with balanced gender distribution (26 male, 24 female), enabling comprehensive cross-case comparisons between high and low performers.

All participants signed informed consent forms before data collection, which detailed the research objectives, procedures, data handling protocols, and their rights. Research data were anonymized through coding, and all original materials will be preserved for 3 years following the study’s completion, after which they will be destroyed. Participants were formally notified that they could withdraw from the study at any time without any impact on their course assessment.

### 3.2 Research procedure

**Figure 2:**
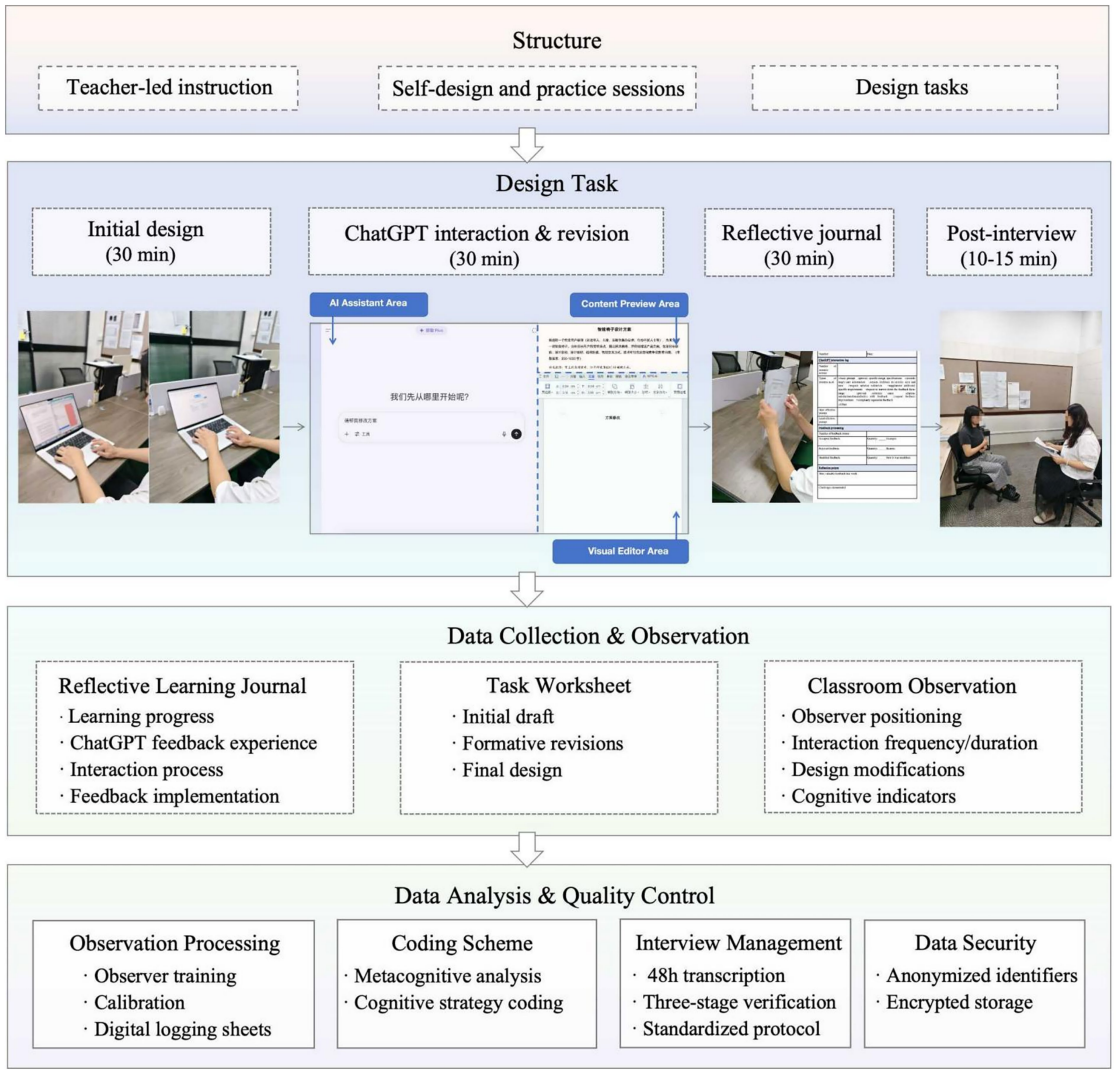
Detailed procedures.

The study was conducted in a single session focusing on a product design task in a laboratory equipped with keystroke logging and screen recording systems. Students were required to develop a multifunctional storage product for learning supplies targeted at university students. The session comprised four sequential phases: initial draft design (30 min), ChatGPT interaction and revision (30 min), reflection journal writing (30 min), and post-task interviews (10–15 min). Participants received standardized written instructions for each phase, ensuring consistency across all sessions while allowing natural variation in ChatGPT interaction strategies during Phase 2. Throughout the design task, students followed these steps: (i) developed an initial written design proposal based on target user needs, including textual descriptions of product positioning, functional features, and design concepts; (ii) obtained design improvement feedback from ChatGPT through purely text-based dialogue, where students described their design ideas in writing and ChatGPT provided textual feedback and suggestions; (iii) revised and refined the written design proposal based on AI feedback; and (iv) submitted the final written design proposal. This text-based interaction approach focused on the conceptualization phase of design thinking, emphasizing how students verbally expressed design intentions and processed AI’s textual feedback. The complete procedure and data collection framework are illustrated in Figure 2.

To capture behavioral engagement patterns, two trained observers documented participants’ design processes using standardized digital logging sheets. The observers completed a training program on observation techniques and documentation protocols to ensure consistency. During observations, they recorded participants’ interaction patterns with ChatGPT, including interaction frequency, duration, and types of prompts used, as well as design modification behaviors and cognitive process indicators. These behavioral data enabled analysis of feedback-seeking strategies and revision patterns that distinguished high from low performers. Observers were positioned at a non-intrusive distance from participants, maintaining consistent positioning and behaviors throughout the study to minimize observer effects. Inter-rater reliability protocols ensured observer consistency across all sessions.

Participants completed reflective learning journals after the ChatGPT interaction phase, recording their learning progress, ChatGPT interaction experiences, and feedback implementation processes. These journals, combined with task worksheets documenting initial drafts, formative revisions, and final outcomes, provided rich data for analyzing cognitive engagement patterns. The documentation formats included text entries, screen captures, audio recordings, and video documentation, allowing us to trace students’ cognitive processing sequences as they navigated between different metacognitive states during AI interaction. Templates for reflective journals and task worksheets were provided to ensure systematic data collection while allowing participants freedom to express their experiences authentically.

Semi-structured interviews were conducted in an acoustically isolated room immediately following the reflection phase to capture participants’ emotional engagement and deeper cognitive processes. The interviews followed a standardized protocol adapted from prior research on AI-assisted learning, exploring five dimensions: operational behaviors with ChatGPT, cognitive processing strategies, emotional responses to AI feedback, challenge and needs encountered, and perceptions of feedback quality. Interviews were recorded with primary and backup digital recording devices and transcribed within 48 h. Transcription accuracy was verified through a three-stage process: initial transcription, interviewer review, and senior researcher verification. This interview data enabled us to understand the emotional dimensions of engagement, including trust, creative confidence, and attitudes toward AI feedback that varied across performance levels.

Data management followed institutional protocols throughout the study. All participants were assigned anonymized identifiers, and data were stored on encrypted secure servers. The comprehensive dataset for each participant included behavioral observation logs, screen recordings, task worksheets showing design evolution, reflective journal entries, and interview transcripts. This multi-source data collection approach enabled triangulation across behavioral, cognitive, and emotional dimensions of engagement during subsequent analysis.

### 3.3 Analysis

The data analysis involved six trained researchers conducting independent coding across three engagement dimensions. Behavioral engagement employed Yan’s (2025) deductive framework for prompt construction strategies [Fleiss’ Kappa *κ* = 0.86, 95% CI (0.78, 0.91)]. Cognitive engagement utilized a deductive framework derived from self-regulated learning theory (Fritzsche et al., 2018), achieving reliable coding of cognitive states [Cohen’s κ = 0.82, 95% CI (0.74, 0.89)] and temporal boundaries [κ = 0.78, 95% CI (0.69, 0.86)]. Emotional engagement followed Braun and Clarke’s (2012) thematic analysis with a mixed deductive-inductive approach. All coders completed systematic training and resolved disagreements through consensus discussion. The following subsections detail specific analytical procedures.

The analytical approach was designed to examine three dimensions of psychological engagement as conceptualized within self-regulated learning theory: behavioral engagement (forethought phase), cognitive engagement (performance phase), and emotional engagement (self-reflection phase) (Zimmerman, 2013). Each dimension was operationalized through specific data sources and analytical methods, forming a theoretically grounded and methodologically rigorous investigation of student-AI interaction patterns.

#### 3.3.1 Research design

Following Yin’s (2018) methodological framework, this study employs an observational multiple-case study design to examine how design students with different performance levels engage with ChatGPT-generated feedback during authentic design tasks. Each participant represents an individual case of AI feedback engagement, analyzed through within-case examination and cross-case comparison between performance groups. This design is observational rather than experimental: we do not manipulate or control students’ use of ChatGPT but rather observe and analyze their naturally occurring interaction patterns in a structured design task context.

Grounded in self-regulated learning theory and educational psychology frameworks, the study examines how performance level relates to three dimensions of student engagement. Performance level was classified based on a comprehensive academic assessment combining design course GPA and technical proficiency, resulting in two groups: high performers (*n* = 25) and low performers (*n* = 25). Behavioral engagement was measured through feedback-seeking patterns (prompt construction strategies, interaction frequency) and revision behaviors (types of design modifications). Cognitive engagement was assessed through sequential patterns of metacognitive and cognitive strategy use during ChatGPT interaction. Emotional engagement was captured through affective responses, attitudes toward AI feedback, and future usage intentions.

Students completed a product design task in which they used ChatGPT to obtain design improvement feedback on their written proposals. This task served as the naturalistic research context for observing engagement patterns, not as an educational intervention being evaluated for effectiveness. All participants had equal access to ChatGPT and received identical task instructions; we imposed no restrictions on how they interacted with the system. This approach enabled observation of authentic variation in engagement strategies across performance levels under comparable conditions.

The choice of multiple case study methodology was justified by three considerations aligned with the exploratory nature of this investigation. First, we prioritized analytical generalization through pattern identification rather than statistical generalization to populations (Yin, 2018), seeking to understand the psychological mechanisms underlying differential engagement with conversational AI systems through in-depth observation of individual interaction processes. Second, case study methodology enabled triangulation of multiple data sources (behavioral observations, cognitive process coding, interview data, and reflective journals) to capture the multidimensional nature of engagement (Gerring, 2004). Third, the sample size of 50 cases (25 per performance group) provided sufficient data for meaningful cross-group comparison while maintaining the depth of analysis characteristic of case study research, an approach consistent with recent AI-assisted learning research employing small-sample intensive designs (Yan and Zhang, 2024).

Following a convergent parallel design (Creswell and Clark, 2017), we collected quantitative behavioral data (interaction frequency, duration, coded behavioral indicators) and qualitative data (semi-structured interviews, observational field notes, reflective journals) simultaneously during the single design session. These data streams were analyzed independently and then integrated during interpretation to provide comprehensive understanding of engagement patterns. Quantitative analysis through descriptive statistics and lag sequential analysis identified behavioral and cognitive patterns, while qualitative thematic coding of interviews revealed the subjective experiences and emotional responses underlying these patterns.

To establish research credibility and trustworthiness, we implemented multiple validation strategies. Data triangulation was achieved across behavioral observations, self-report measures, and interview data. Investigator triangulation was established through dual coding of behavioral sequences and cognitive states, with disagreements resolved through discussion. Member checking involved presenting preliminary findings to participants for verification of interpretative accuracy. Peer debriefing with domain experts occurred throughout the data analysis process. Finally, we maintained detailed audit trail documentation of all research procedures and analytical decisions. These measures enhanced both internal validity (credibility of interpretations) and external validity (transferability to similar contexts).

#### 3.3.2 Data-to-variable mapping and analytical framework

Following established educational psychology frameworks (Reeve et al., 2020), each engagement dimension was systematically mapped to specific data sources collected during the study. This operationalization followed a hierarchical measurement framework that systematically progressed from theoretical dimensions to observable behavioral indicators: each dimension was decomposed into specific measurable elements, linked to concrete behavioral measures, assessed through standardized data collection protocols, and coded using validated analytical frameworks.

Behavioral engagement was assessed through observational field notes and task worksheets documenting feedback-seeking strategies (prompt construction patterns, interaction frequency) and revision behaviors (types of design modifications). Cognitive engagement was examined through sequential analysis of behavioral observations, reflective learning journals, and task worksheets showing cognitive and metacognitive strategy deployment. Emotional engagement was captured through semi-structured interview transcripts revealing affective responses, attitudes toward AI feedback, and future usage intentions.

The emotional engagement interviews employed a standardized protocol with five core questions designed to systematically probe participants’ affective experiences and cognitive processes: (Q1) “How did you feel when receiving and processing ChatGPT’s feedback? Can you describe specific moments that triggered positive or negative emotions?”; (Q2) “How would you describe your overall interaction experience with ChatGPT during this design task? What aspects enhanced or hindered your learning?”; (Q3) “What challenges or difficulties did you encounter when using ChatGPT for design feedback? What support or guidance did you need?”; (Q4) “What strategies or approaches did you use to make effective use of ChatGPT’s suggestions? How did you decide which feedback to accept or reject?”; and (Q5) “Based on this experience, how likely are you to continue using ChatGPT for future design work? What factors influence this decision?”

This multi-source triangulation enabled comprehensive examination of psychological engagement across all three theoretical dimensions. Detailed specifications of behavioral coding categories, cognitive state definitions, and emotional thematic frameworks are presented in the following subsections (3.3.3–3.3.5), with complete operational definitions and coding schemes provided in Appendices C, D.

#### 3.3.3 Behavioral engagement analysis

Behavioral engagement analysis employed quantitative document analysis of observational logs and task worksheets. The analytical framework was theoretically grounded in self-regulated learning theory’s forethought phase, which emphasizes strategic planning and goal-oriented behavior (Zimmerman, 2013). Two specific behavioral indicators were quantified: feedback-seeking patterns and revision behaviors.

For feedback-seeking behaviors, the coding scheme was adapted deductively from Yan’s (2025) framework for analyzing prompt construction in ChatGPT interactions, modified to account for design education contexts. The framework categorizes prompts into 11 types: basic prompt (BP), providing specific design specifications [+SPE], including target user information [+TAR], requesting feedback in specific style and tone [+TON], seeking solution validation [+VAL], supplementing additional requirements [+ADD], requesting narrowed feedback focus [-NAR], providing reference cases [+REF], expressing satisfaction/dissatisfaction [+EXP], requesting feedback improvement [+IMP], and completely regenerating feedback [!REV]. These categories were deductively derived from prior research on human-AI interaction in creative contexts, reflecting theoretically established dimensions of strategic feedback-seeking behavior.

Two trained coders from the research team independently analyzed all participant data using standardized digital coding sheets. Prior to analysis, coders completed a systematic training protocol involving practice coding of pilot data, discussion of coding rules, and calibration sessions to ensure consistent application of the framework. Inter-rater reliability achieved satisfactory agreement [Fleiss’ Kappa *κ* = 0.86, 95% CI (0.78, 0.91)]. Discrepancies were resolved through collaborative discussion and consensus-building. Behavioral data were analyzed using descriptive statistics (means, standard deviations, frequency distributions) to identify patterns distinguishing high from low performers, consistent with exploratory case study methodology prioritizing pattern identification over statistical inference.

#### 3.3.4 Cognitive engagement analysis

Cognitive engagement analysis employed lag sequential analysis (LSA) to examine transition patterns between cognitive and metacognitive states, grounded in educational psychology research on metacognitive monitoring and regulation (Fritzsche et al., 2018). This analytical approach aligns with self-regulated learning theory’s performance phase, which emphasizes strategy implementation and metacognitive monitoring during task execution.

Behavioral units were defined based on participants’ continuous ChatGPT interactions, with each unit representing a discrete cognitive action having clear temporal and operational boundaries (minimum 3 s, maximum 60 s). Unit boundaries were determined by: (i) completion of coherent cognitive operations, (ii) observable transitions in focus or activity visible in screen recordings, and (iii) natural breakpoints in cognitive flow identified through verbal protocols.

The cognitive coding framework employed six deductively derived categories grounded in educational psychology literature on feedback processing and metacognition (see Appendix D for detailed operational definitions): purpose statement (P), representing clear articulation of design objectives; new design query (Q), indicating specific design-related questions posed to ChatGPT; feedback processing (R), representing reading and reviewing ChatGPT’s responses; modification interaction (M), showing requests for modifications to ChatGPT’s suggestions; design decision (D), indicating clear decisions regarding ChatGPT’s suggestions; and implementation decision-making (I), representing incorporation of confirmed modifications into designs. These categories reflect established psychological constructs in self-regulated learning research, specifically metacognitive planning (P), information seeking (Q), comprehension monitoring (R), strategy evaluation (M, D), and behavioral execution (I).

Two trained researchers independently coded all behavioral sequences using this framework. To ensure coding reliability, both coders completed a training phase using 20% of randomly selected participant sessions, achieving satisfactory inter-rater reliability for cognitive state identification [Cohen’s *κ* = 0.82, 95% CI (0.74, 0.89)] and temporal boundary identification [κ = 0.78, 95% CI (0.69, 0.86)]. Disagreements were resolved through discussion until consensus was reached.

Lag sequential analysis was implemented through GSEQ 5.1 software (Bakeman et al., 2005), calculating adjusted residuals of transition probability matrices to identify significant sequential patterns. The statistical significance of behavioral transitions was determined by *Z*-scores of adjusted residuals, with values exceeding 1.96 indicating significant transitions at *p* < 0.05. These transition patterns were visualized through network diagrams to illustrate cognitive processing sequences characteristic of high versus low performers.

#### 3.3.5 Emotional engagement analysis

Emotional engagement analysis employed Braun and Clarke’s (2012) six-step thematic analysis method on semi-structured interview transcripts. This approach aligns with self-regulated learning theory’s self-reflection phase, which emphasizes affective responses and their influence on future learning engagement (Linnenbrink-Garcia et al., 2016).

The analytical framework employed a mixed deductive-inductive coding approach. The initial coding framework was deductively derived from established psychological constructs in educational psychology: emotional responses to AI feedback (grounded in research on affective learning experiences) (Heilporn et al., 2021); design learning experiences (based on design cognition literature) (Cross, 2004); encountered challenges and needs (informed by cognitive load theory) (Feng, 2025); learning strategy applications (derived from self-regulated learning frameworks) (Zimmerman, 2013); and future usage intentions (based on technology acceptance research) (Pan, 2023). Within each deductive category, specific subcategories emerged inductively from the data through iterative coding and theme development.

The complete hierarchical coding framework, including detailed categorization rules and exemplar quotes, is provided in Appendix C. This framework captures five primary dimensions: (i) emotional responses (ER), including positive affect, negative affect, and mixed emotional states; (ii) design learning experiences (DE), encompassing perceived productivity, exploratory value, and feedback quality perceptions; (iii) encountered challenges and needs (CN), covering cognitive load, prompt engineering difficulties, and psychological pressure; (iv) learning strategy applications (LS), reflecting adaptive behaviors and metacognitive adjustments; and (v) future usage intentions (FI), indicating continuance intentions and anticipated usage patterns.

Two additional researchers from the team conducted independent coding following Braun and Clarke’s (2012) systematic procedure: familiarization with data through repeated reading of transcripts, generation of initial codes, searching for themes, reviewing themes for internal coherence and external distinctiveness, defining and naming themes, and producing the final analysis. Regular research meetings facilitated comparison of coding results, resolution of discrepancies through collaborative discussion, and establishment of consensus on theme boundaries and interpretations. This iterative process ensured analytical rigor while remaining responsive to emergent patterns in the data.

#### 3.3.6 Data integration and triangulation

Following the convergent parallel mixed-methods design (Creswell and Clark, 2017), quantitative behavioral data and qualitative interview data were analyzed independently and then integrated during interpretation. Triangulation across multiple data sources (behavioral observations, cognitive process coding, reflective journals, interview transcripts) and multiple analytical methods (descriptive statistics, lag sequential analysis, thematic analysis) enhanced the credibility and trustworthiness of findings. Convergence and divergence across data sources were systematically examined to provide comprehensive understanding of psychological engagement patterns, with particular attention to how behavioral, cognitive, and emotional dimensions interrelate within the theoretical framework of self-regulated learning.

## 4 Results

### 4.1 Behavioral engagement

The quantitative document analysis yielded data regarding two aspects of participant engagement: feedback seeking and revision processes. The feedback-seeking behavior manifests in participants’ formulation of ChatGPT prompts, while the revision behavior is evidenced in their processing of ChatGPT’s feedback across various error types.

Drawing on the coding scheme adapted from Yan (2025), the analysis revealed several distinct types of feedback-seeking behaviors: basic prompt (BP); providing specific design specifications [+SPE]; including target user information [+TAR]; requesting feedback in specific style and tone [+TON]; seeking solution validation [+VAL]; supplementing additional specific requirements [+ADD]; requesting to narrow down the feedback focus range [−NAR]; providing reference cases [+REF]; expressing satisfaction/dissatisfaction with feedback [+EXP]; requesting feedback improvement [+IMP]; and completely regenerating feedback [!REV].

**Figure 3:**
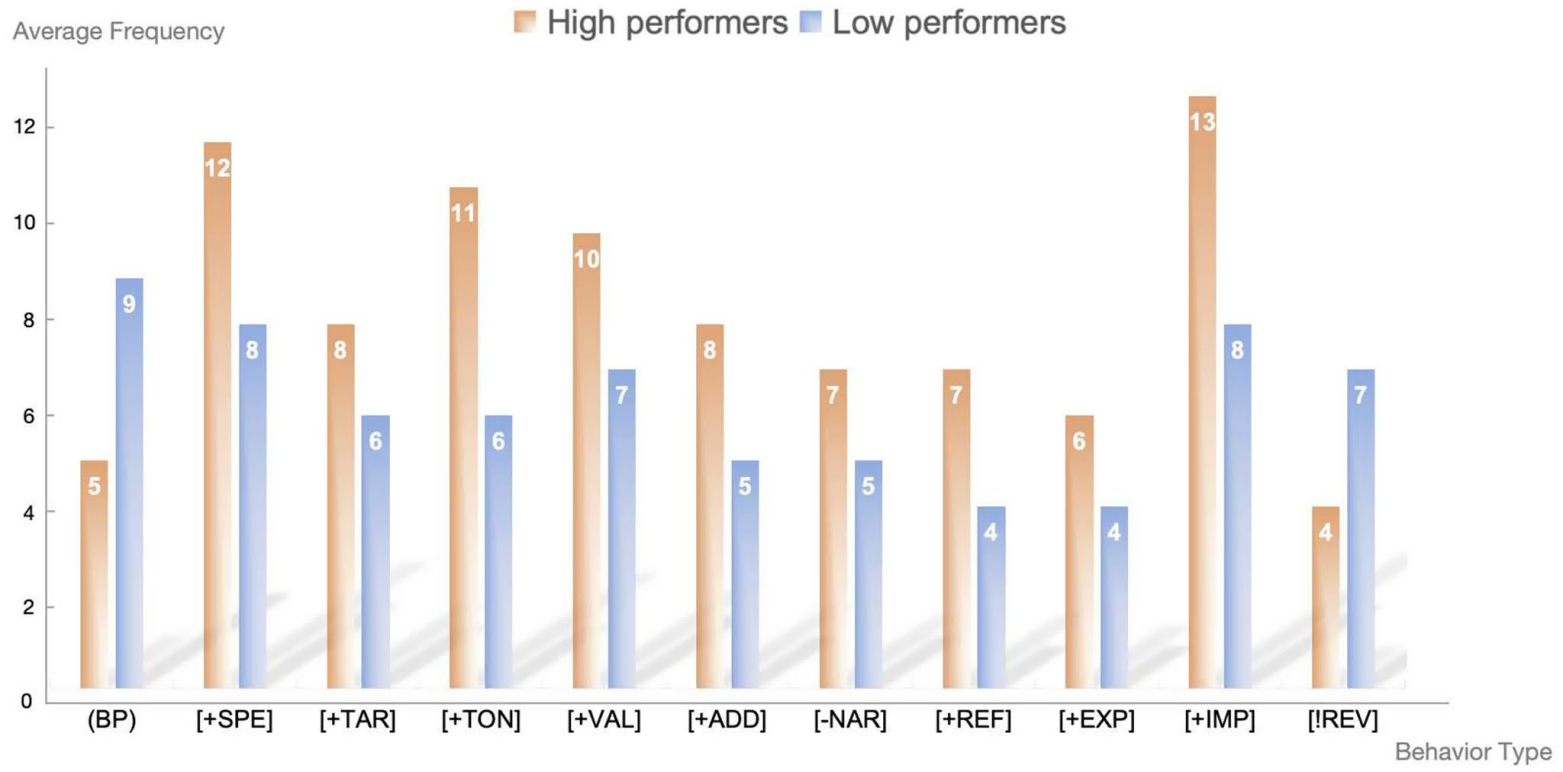
Feedback-seeking behaviors (average frequencies).

As shown in Figure 3, notable differences emerged between high performers (*n* = 25) and low performers (*n* = 25) in their feedback-seeking behaviors during the single design session. High performers demonstrated more sophisticated and diverse engagement patterns compared to their low-performing counterparts.

High performers exhibited substantially higher frequencies in advanced feedback-seeking behaviors. Specifically, they showed greater use of specification behaviors (+SPE: *M* = 18.2, *SD* = 4.1 vs. *M* = 11.3, *SD* = 3.2), improvement-seeking behaviors (+IMP: *M* = 16.8, *SD* = 3.9 vs. *M* = 9.7, *SD* = 2.8), and validation-seeking behaviors (+VAL: *M* = 14.5, *SD* = 3.2 vs. *M* = 8.1, *SD* = 2.5). Additionally, high performers more frequently requested feedback in specific styles and tones (+TON: *M* = 12.3, *SD* = 2.8 vs. *M* = 6.9, *SD* = 2.1) and provided reference cases (+REF: *M* = 8.7, *SD* = 2.3 vs. *M* = 4.2, *SD* = 1.8).

Conversely, low performers showed greater reliance on basic prompts (BP: *M* = 15.2, *SD* = 4.3 vs. *M* = 6.8, *SD* = 2.9), indicating a more straightforward approach to ChatGPT interaction. While both groups utilized target user information (+TAR) and additional requirements (+SPE), high performers demonstrated substantially more frequent and strategic application of these behaviors.

The analysis of revision behaviors revealed distinct patterns between performance groups. High performers demonstrated more sophisticated feedback processing strategies, characterized by alternative modifications that refined and enhanced their design solutions. Their revision patterns indicated systematic evaluation and selective implementation of ChatGPT’s suggestions, with greater emphasis on iterative improvement.

Low performers, in contrast, exhibited more conservative revision approaches, primarily employing deletional modifications. The conservative revision approach suggested uncertainty in processing AI feedback. Their revision behaviors indicated less confidence in evaluating and implementing complex feedback suggestions.

High performers demonstrated superior capabilities in prompt engineering, consistently providing detailed contextual information and specific requirements in their interactions with ChatGPT. Their prompts were characterized by clarity of design objectives, comprehensive user requirement specifications, and explicit requests for particular types of feedback.

Low performers tended to rely on more basic prompt structures, often lacking sufficient contextual enrichment or specific guidance for the AI system. The difference in prompt construction quality directly influenced the relevance and applicability of the feedback received, creating a cycle where more sophisticated prompting led to more valuable feedback and subsequent design improvements.

### 4.2 Cognitive engagement

Tables 2, 3 present the lag sequential analysis (LSA) results for participants. In these tables, the leftmost column represents the initial behavior, while the top row represents the subsequent behaviors in the sequence. To analyze participants’ cognitive engagement patterns, data were coded into six categories: purpose statement (P), referring to clear statements of design objectives; new design query (Q), indicating specific design-related questions posed to ChatGPT; feedback processing (R), representing the reading and reviewing of ChatGPT’s responses; modification interaction (M), showing requests for modifications to ChatGPT’s suggestions; design decision (D), indicating clear decisions made regarding ChatGPT’s suggestions; and implementation decision making (I), representing the incorporation of confirmed modifications into the design (Appendix D). The adjusted residuals serve as the criterion, wherein Z-values exceeding 1.96 indicate significant correlations in the behavioral sequence.

**Table 2:** LSA of high performers (Frequencies and adjusted residuals).

From/To	P	Q	R	M	D	I
P	4 (−7.90)	**116 (11.28)** ^*^	**84 (4.37)** ^*^	25 (−3.90)	3 (−5.25)	9 (−1.10)
Q	52 (0.77)	0 (−9.26)	**135 (12.84)** ^*^	44 (−0.49)	11 (−3.41)	2 (−3.44)
R	37 (−2.79)	55 (−0.56)	9 (−9.32)	**134 (14.59)** ^*^	34 (1.35)	2 (−3.74)
M	**64 (4.18)** ^*^	40 (−1.19)	35 (−3.00)	3 (−7.35)	**64 (10.33)** ^*^	9 (−0.69)
D	**41 (4.72)** ^*^	13 (−2.75)	8 (−4.47)	9 (−3.18)	6 (−1.87)	**36 (13.60)** ^*^
I	**24 (5.06)** ^*^	**21 (3.48)** ^*^	4 (−2.79)	2 (−2.83)	0 (−2.49)	0 (−1.70)
^*^Z > 1.96						

*n* = 25. P = Purpose statement (clear statements of design objectives); Q = Query (specific design-related questions posed to ChatGPT); R = Feedback processing (reading and reviewing ChatGPT’s responses); M = Modification interaction (requests for modifications to ChatGPT’s suggestions); D = Design decision (clear decisions made regarding ChatGPT’s suggestions); I = Implementation (incorporation of confirmed modifications into the design). Bold values with asterisks (*) indicate statistically significant transitions (*Z* > 1.96).

**Table 3:** LSA of low performers (Frequencies and adjusted residuals).

From/To	P	Q	R	M	D	I
P	3 (−7.61)	**69 (4.59)** ^*^	19 (−3.31)	30 (−1.62)	20 (−1.01)	**86 (8.43)** ^*^
Q	**68 (5.14)** ^*^	0 (−8.14)	**89 (11.57)** ^*^	40 (0.68)	11 (−3.00)	10 (−5.91)
R	23 (−1.97)	18 (−3.22)	2 (−5.60)	**70 (9.24)** ^*^	**43 (6.72)** ^*^	14 (−3.75)
M	**52 (3.71)** ^*^	20 (−3.14)	16 (−2.66)	3 (−5.94)	**44 (6.55)** ^*^	**45 (2.45)** ^*^
D	20 (−0.46)	10 (−3.11)	4 (−3.75)	**31 (3.08)** ^*^	0 (−3.91)	**50 (7.28)** ^*^
I	44 (1.33)	**100 (12.15)** ^*^	**41 (2.30)** ^*^	12 (−4.43)	0 (−5.35)	0 (−7.38)
^*^Z > 1.96						

*n* = 25. P, Purpose satement (clear statements of design objectives); Q = Query (specific design-related questions posed to ChatGPT); R = Feedback processing (reading and reviewing ChatGPT’s responses); M = Modification interaction (requests for modifications to ChatGPT’s suggestions); D = Design decision (clear decisions made regarding ChatGPT’s suggestions); I = Implementation (incorporation of confirmed modifications into the design). Bold values with asterisks (*) indicate statistically significant transitions (*Z* > 1.96).

About high performer analysis (Table 2), the sequential analysis of the high performers identified several significant cognitive transitions. The data revealed strong transitions from purpose statements to queries (P → Q, *Z* = 11.28) and feedback processing (P → R, *Z* = 4.37). The most prominent pattern was the transition from queries to feedback processing (Q → R, *Z* = 12.84), indicating systematic information-seeking behavior. Subsequent cognitive processes included significant transitions from feedback processing to modifications (R → M, *Z* = 14.59), representing the most frequent post-feedback processing activity. The sequence continued with transitions from modifications to design decisions (M → D, *Z* = 10.33) and from design decisions to implementation (D → I, *Z* = 13.60). Additionally, the high performer demonstrated cyclical engagement patterns, with significant transitions from modifications back to purpose statements (M → P, *Z* = 4.18), from design decisions back to purpose statements (D → P, *Z* = 4.72), and from implementation to both purpose statements (I → P, *Z* = 5.06) and queries (I → Q, *Z* = 3.48).

About low performer analysis (Table 3), the low performers’ sequential analysis showed distinctly different cognitive patterns. Key significant transitions included movements from purpose statements to queries (P → Q, *Z* = 4.59) and from purpose statements directly to implementation (P → I, *Z* = 8.43), suggesting a more direct approach to design execution. The analysis revealed significant transitions from queries to both purpose statements (Q → P, *Z* = 5.14) and feedback processing (Q → R, *Z* = 11.57). Feedback processing activities showed significant transitions to both modifications (R → M, *Z* = 9.24) and design decisions (R → D, *Z* = 6.72). The low performer also demonstrated significant transitions from modifications to purpose statements (M → P, *Z* = 3.71), design decisions (M → D, *Z* = 6.55), and implementation (M → I, *Z* = 2.45). A notable pattern was the transition from design decisions to implementation (D → I, *Z* = 7.28) and from implementation back to queries (I → Q, *Z* = 12.15) and feedback processing (I → R, *Z* = 2.30).

The comparison between high and low performers reveals distinct cognitive engagement strategies. The high performer demonstrated more systematic sequential processing, with stronger emphasis on query-to-feedback-to-modification chains (Q → R → M), indicating methodical information processing. In contrast, the low performer showed more direct pathways from purpose statements to implementation (P → I, *Z* = 8.43), suggesting a tendency toward immediate action rather than comprehensive feedback processing. The high performer’s pattern of returning to purpose statements from multiple stages (M → P, D → P, I → P) indicates more reflective and iterative thinking, while the low performer’s strong implementation-to-query transition (I → Q, *Z* = 12.15) suggests a more reactive approach to seeking additional information after implementation attempts.

**Figure 4:**
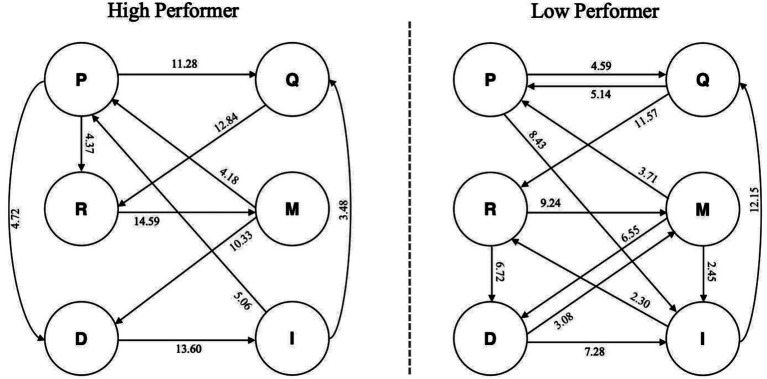
Cognitive engagement transformation diagram of the participants.

Figure 4 shows the visualization of participants’ LSA. In the figure, each node represents a type of cognitive or metacognitive strategy, and the connections between nodes indicate statistically significant transitional relationships in the cognitive sequences.

### 4.3 Emotional engagement

Analysis of in-depth interviews with 50 participants revealed their emotional engagement experiences during the ADIF process using ChatGPT (Table 4). The analysis of emotional engagement required a sophisticated multi-dimensional coding framework, encompassing emotional responses (ER), design experiences (DE), encountered challenges (CN), design strategy applications (LS), and future usage intentions (FI). The complexity and interconnected nature of emotional engagement in design practice environments necessitated this comprehensive analytical approach, as emotional responses in design learning are inherently intertwined with cognitive processes and behavioral manifestations. A detailed hierarchical coding scheme was developed to capture these intricate relationships (see Appendix C for the complete coding framework and detailed categorization rules). The findings emerged across three dimensions: (i) participants’ perception of the experience as a productive design process (coded primarily through DE-P); (ii) participants acknowledged challenges and psychological pressure during usage (coded through CN-C and ER-M), while demonstrating higher emotional acceptance of AI feedback compared to teacher feedback; and (iii) participants expressed positive intentions for continued use (coded through FI-C).

**Table 4:** Emotional engagement patterns: Frequencies and percentages (*n* = 50).

Dimension	Specific manifestation	*n*	%
Positive learning experience	Overall positive evaluation	47	94%
	Emphasized exploratory value	38	76%
	Acknowledged varying feedback quality	42	84%
	Valued immediate and automated feedback	45	90%
Cognitive load and psychological pressure	Invested effort in optimizing prompts	35	70%
	Experienced cognitive load	32	64%
	Felt more relaxed learning atmosphere	28	56%
	Required time for filtering feedback	31	62%
	Perceived AI interaction as more efficient	44	88%
Future usage intention	Will continue using ChatGPT	48	96%
	To avoid pressure from traditional feedback	29	58%
	Plan to explore advanced features	41	82%
	Reported difficulties in prompt expression	17	34%

First, participants expressed predominantly positive evaluations of the learning process using ChatGPT for ADIF (*n* = 47, 94%). High performers demonstrated particularly strong recognition of ChatGPT’s design feedback capabilities. Participants characterized their experience as a progressive design journey, emphasizing its exploratory value (*n* = 38, 76%). While maintaining positive attitudes toward the learning environment, participants retained objectivity regarding their personal progress. Participants acknowledged the experience as beneficial, while noting the varying quality of ChatGPT’s feedback (*n* = 42, 84%). Participants emphasized ChatGPT’s advantages in immediate response and automated feedback (*n* = 45, 90%), consistently highlighting the professionalism and practical value of the feedback.

Second, the analysis revealed a noteworthy cognitive phenomenon: despite ChatGPT’s ability to provide continuous feedback and suggestions, participants invested considerable effort in contemplating and optimizing prompts (*n* = 35, 70%). This phenomenon likely stemmed from the pursuit of high-quality AI feedback; although ChatGPT could assist with ideation, a consistent pattern of refining the interaction process emerged among participants. While this increased self-demand demonstrated learning engagement, it potentially introduced additional cognitive load (*n* = 32, 64%). Conversely, participants indicated that interactions with ChatGPT fostered a more relaxed learning atmosphere (*n* = 28, 56%). This psychological comfort derived from AI’s non-judgmental nature—eliminating concerns about criticism for inappropriate expressions or incorrect viewpoints. Participants received effective feedback but observed that ChatGPT’s comprehensive and rich feedback necessitated additional time for filtering and integration (*n* = 31, 62%). Participants emphasized the efficiency of AI interaction (*n* = 44, 88%), noting that compared to traditional search engines, it reduced the cognitive load required for information filtering.

The findings indicated that participants expressed willingness to continue using ChatGPT (*n* = 48, 96%), reflecting that artificial intelligence-driven feedback’s practical value in interactive design environments is receiving recognition from students. Participants observed the future potential of AI in design feedback, with one high-performing participant noting: “Using AI for revision feedback will become standard practice in the future, and the techniques and tips we are exploring now will have pioneering significance.” Participants indicated they would continue using ChatGPT after the project to avoid potential pressure from traditional face-to-face teacher feedback (*n* = 29, 58%). Participants demonstrated learning initiative, planning to further explore ChatGPT’s advanced features in design courses (*n* = 41, 82%). However, the technological application presented challenges; participants reported difficulties in prompt expression when interacting with ChatGPT, leading to frequent generation of redundant content (*n* = 17, 34%), suggesting the necessity for additional time investment in developing relevant skills.

Performance-level differences were evident in emotional engagement patterns. High performers (*n* = 25) generally approached ChatGPT more as a “dialogue partner” for design inspiration, demonstrating greater emotional investment and more sophisticated interaction patterns. In contrast, low performers (*n* = 25) tended to view ChatGPT more as a “guide,” showing more cautious emotional engagement but still maintaining positive attitudes toward the technology. These differences in emotional positioning influenced their overall depth of engagement with the AI feedback system, though both groups showed strong intentions for continued use.

## 5 Discussion

### 5.1 Feedback-seeking and revision Behaviors

The observation revealed a common developmental trajectory in participants’ feedback-seeking behaviors: all showed progressive adaptation to ChatGPT’s feedback mechanism, transitioning from simple interactions to more sophisticated engagement patterns over time. This evolutionary pattern reflects a natural learning curve in adapting to AI-assisted design environments, regardless of performance level. From a technological interaction perspective, high performers exhibited stronger capabilities in utilizing ChatGPT’s functional features strategically (Yan and Wang, 2022). The framework helps explain why high performers achieved more substantive behavioral engagement in the feedback process, particularly in their ability to construct effective prompts and iterate through feedback cycles. From a feedback processing standpoint, the feedback utilization theory proposed by Narciss et al. (2022) suggests that individuals develop different strategies for processing and implementing external feedback. This theoretical lens helps illuminate why high performers demonstrated superior capability in translating ChatGPT’s feedback into specific design improvements.

These strategic differences manifested in students’ prompt construction and feedback implementation approaches. High performers demonstrated more sophisticated prompt formulation strategies, focusing on providing detailed contextual information and specific requirements. In contrast, low performers tended to rely on basic prompts without sufficient contextual enrichment. This aligns with recent findings that suggest proficiency levels significantly influence students’ ability to effectively communicate their needs to AI systems (Adeshola and Adepoju, 2024).

The revision behavior patterns require methodological clarification. Our characterization of high performers’ alternative modifications and low performers’ deletional modifications was derived from systematic observation of students’ revision processes during the ChatGPT interaction phase rather than analysis of final design outputs. Trained observers documented each revision action, coding whether students (i) replaced existing design elements with alternatives, (ii) removed elements without replacement, or (iii) added new elements. High performers exhibited significantly higher frequencies of alternative modifications compared to low performers, suggesting more evaluative and iterative engagement with feedback. However, we acknowledge this observational approach cannot definitively establish whether these behavioral differences translated into superior final design quality.

Particularly regarding revision behavior patterns, the research identified distinct variations: high performers were inclined towards alternative modifications, while low performers preferred deletional modifications. This distinction manifested not merely in modification frequency but further illustrated divergent feedback processing strategies amongst individuals of varying skill levels. High performers refined design solutions through active alternative modifications (Yan and Zhang, 2024), whereas low performers’ deletional modifications indicated their uncertainty and conservative approach towards ChatGPT feedback. The modification behavior pattern observed in the design field reveals the crucial value of alternative modifications in the evolution and refinement of design solutions. The finding aligns with Thorp’s (2023) assertion that in AI-assisted design environments, there exists a specific need to enhance low performers’ feedback application capabilities.

### 5.2 Metacognitive regulation patterns

The sequential analysis revealed that students demonstrated significant differences in metacognitive regulation, particularly manifested in their processing of design feedback provided by ChatGPT. High performers exhibited more systematic and complete metacognitive regulation patterns, specifically showing effective planning, monitoring, and evaluation capabilities. In contrast, low performers tended to adopt simple linear thinking patterns, often overlooking critical feedback evaluation phases. The disparity in metacognitive regulation capabilities can be examined through two dimensions. Firstly, from a cognitive processing perspective, Halkiopoulos and Gkintoni (2024) point out that limitations in individual cognitive abilities affect their adaptive performance in AI-assisted environments. These findings align with those of Fritzsche et al. (2018), which suggest that individual basic cognitive abilities influence their performance in automated feedback environments. Particularly in design, a field requiring multidimensional thinking, differences in cognitive abilities lead to significant variations in feedback processing quality.

However, from a technical interaction perspective, our findings offer novel perspectives. Notably, even individuals with limited design proficiency could partially compensate for their metacognitive monitoring deficiencies through iterative prompt refinements (Sato and Loewen, 2018). As a conversational AI system, ChatGPT provides personalized support for individuals at different levels through iterative interactions (Wu et al., 2023). This interactive characteristic effectively creates a scaffolded learning environment, enabling low performers to gradually establish basic metacognitive frameworks. The finding corroborates Lo et al.’s (2024) research, demonstrating that AI-driven design feedback systems can promote the development of metacognitive abilities through adaptive dialogue.

From a more fundamental perspective, these differences in metacognitive regulation patterns may suggest diverse cognitive strategy preferences when encountering AI feedback. The analysis suggests that high performers might be more inclined to approach ChatGPT as a tool for cognitive development, potentially using it to explore design problems, examine various solutions, and integrate different feedback elements. Low performers, on the other hand, appeared to adopt more straightforward interaction patterns, which could indicate a tendency to view ChatGPT primarily as an information source. These observed differences in approach might contribute to varying levels of feedback processing depth and application effectiveness, though further research would be needed to establish more definitive connections between strategy selection and learning outcomes.

### 5.3 Emotional engagement in AI-assisted design environment

Interview data showed that participants exhibited generally positive attitudes towards ChatGPT-generated ADIF. They also responded favourably to the new AI-driven design environment. The high level of recognition and emotional engagement with ChatGPT as a feedback provider corresponds to recent research findings (Koltovskaia et al., 2024). These positive attitudes align with studies on Chinese university students, where trust and perceived usefulness emerged as significant factors influencing ChatGPT adoption (Liu and Zhang, 2024; Shahzad et al., 2024). Specifically, participants unanimously agreed that ChatGPT could provide professional and practical design suggestions (Lo et al., 2024). Compared to traditional ADE systems, ChatGPT received more positive evaluations regarding feedback quality and interaction experience (Steiss et al., 2024).

Moreover, deeper analysis revealed a distinct duality in participants’ emotional engagement. First, regarding cognitive load, participants reflected on the psychological pressure of using ChatGPT for design, specifically concerning the mental burden of processing and integrating design feedback. Such pressure corresponds to findings of Feng (2025), which indicate that AI-assisted feedback systems often increase individual psychological burden. It can be attributed to ChatGPT’s conversational characteristics providing individuals with greater sense of control and autonomy, which partially counteracts the negative effects of cognitive load (Chen et al., 2025). Second, unique contradictions emerged regarding emotional security. Participants generally reported feeling less evaluative pressure when interacting with ChatGPT, forming a marked contrast with traditional teacher-student feedback scenarios. However, they also expressed concerns about AI feedback potentially affecting design originality. This contradictory psychological state between obtaining professional guidance and maintaining creative autonomy provides new empirical evidence for understanding the concept of “creativity anxiety in AI-assisted design” proposed by Magni et al. (2024).

The findings reveal potential differences in emotional engagement patterns between high and low performers. High performers appeared to approach ChatGPT more as a “dialogue partner” for design inspiration, while low performers seemed to view it more as a “guide.” Such differences in perspective might influence their depth of emotional investment and feedback receptivity. These results challenge the previously prevalent view in research that individual emotional responses to AI feedback primarily depend on system technical characteristics (Kocoń et al., 2023). It suggests that individual ability levels and cognitive frameworks may play a role in shaping their emotional experiences. Future AI-assisted design environments might benefit from providing differentiated emotional support mechanisms while maintaining feedback professionalism (Rezai et al., 2024).

### 5.4 Theoretical contributions and future research directions

The findings extend self-regulated learning theory to conversational AI contexts in three ways. First, effective ChatGPT engagement requires metacognitive awareness of both design thinking and AI system requirements, representing a dual metacognitive demand absent in traditional feedback mechanisms. This suggests AI literacy in creative domains involves metacognitive sophistication in translating design intentions into machine-interpretable prompts rather than merely technical competence. Second, performance-level differences in engagement patterns suggest conversational AI systems may inadvertently amplify existing educational inequalities, challenging assumptions about AI’s democratizing potential in education. High performers’ superior metacognitive capabilities enable greater value extraction from AI interactions, potentially widening performance gaps. Third, while ChatGPT’s conversational nature theoretically offers scaffolding for less skilled learners, low performers struggle to exploit this affordance without existing metacognitive foundations, suggesting interventions should focus on metacognitive skill development rather than assuming AI self-evidently benefits all learners.

For pedagogical practice, the findings suggest three implications. First, AI feedback systems should incorporate differentiated scaffolding with metacognitive prompts. Such prompts can encourage high performers to articulate design reasoning while providing structured templates to help low performers formulate effective queries. Second, teachers should reposition their roles from primary feedback providers to metacognitive coaches who help students develop effective AI interaction strategies. This includes explicit instruction in prompt engineering and critical evaluation of AI suggestions. Third, institutions should establish clear expectations about AI’s role as a thinking tool rather than a replacement for human creativity. AI literacy components could be included in design curricula. These implications may hold particular relevance for Chinese design education contexts. Students in this study reported reduced evaluative pressure when interacting with ChatGPT. The finding suggests that AI feedback systems may offer distinctive affordances in educational cultures where traditional teacher-student hierarchies can create barriers to feedback-seeking. Furthermore, the performance-level disparities identified in this study highlight implementation considerations for institutions seeking to integrate AI feedback tools equitably across diverse student populations.

Future research should address several directions emerging from this study. First, longitudinal investigations tracking students across multiple design projects would illuminate how engagement strategies develop over time and whether low performers’ linear patterns evolve toward high performers’ cyclical patterns. Second, research integrating final design output analysis with process observation would establish whether behavioral and cognitive differences translate into superior design quality. Third, investigations examining cognitive flexibility and digital competencies as potential moderators of performance-level differences would advance understanding of individual difference factors shaping AI engagement effectiveness. Fourth, cross-cultural validation across diverse institutional settings, academic levels, and design specializations would establish broader applicability of these findings. These directions align with emerging research on cognitive flexibility, self-regulation skills, and GAI acceptance in educational contexts.

## 6 Conclusion and limitations

### 6.1 Conclusion

This multiple case study reveals how performance-level differences systematically shape psychological engagement with ChatGPT-generated design feedback across behavioral, cognitive, and emotional dimensions. The study’s primary contribution lies in extending self-regulated learning theory to conversational AI contexts in creative education. While existing research examines AI acceptance and behavioral patterns in aggregate, this investigation uncovers the metacognitive mechanisms distinguishing effective from ineffective AI engagement. High performers treat ChatGPT as a collaborative thinking partner through cyclical metacognitive monitoring, while low performers adopt linear, guidance-seeking approaches reflecting limited metacognitive regulation. Critically, these differences suggest conversational AI systems may amplify rather than reduce educational inequalities without appropriate metacognitive scaffolding, challenging assumptions about AI’s democratizing potential.

For practice, the findings demonstrate that effective AI-assisted learning requires psychologically-informed design. Simply providing access to ChatGPT suggests insufficient; students need differentiated scaffolding supporting metacognitive development, explicit instruction in critical evaluation of AI’s epistemological limitations, and teacher guidance repositioned toward metacognitive coaching rather than primary feedback provision.

This exploratory study offers initial insights into how performance-level differences influence psychological engagement with ChatGPT-generated feedback in design education. The findings suggest that further investigation is needed to understand how these engagement patterns develop over time, how they relate to actual design outcomes, and what individual factors may moderate their effectiveness. Such investigations would contribute to ongoing discussions about designing equitable and effective AI-assisted learning environments in creative education.

### 6.2 Limitations

Several limitations of the current investigation should be addressed. First, the study was conducted within a single institutional context with design students. The limitation should be understood within the framework of analytical rather than statistical generalization. The theoretical insights regarding differential engagement patterns between high and low performers are likely transferable to similar design education contexts, though specific manifestations may vary across institutions, cultures, and disciplines. Future research should examine these patterns across diverse institutional settings, academic levels (graduate vs. undergraduate), and design specializations (graphic design, industrial design, UX/UI design) to establish broader applicability. Additionally, cross-cultural validation would strengthen understanding of how cultural factors influence AI engagement patterns in design education environments.

Second, as an observational case study, this research describes engagement patterns and identifies associations between performance levels and engagement strategies rather than establishing causal relationships. While this design allows for rich, contextualized understanding of naturally occurring human-AI interaction patterns, it limits our ability to make causal claims about how performance level influences engagement behaviors. Future research could strengthen causal inference by employing quasi-experimental designs with randomized group assignment and controlled AI feedback conditions. Such designs would enable more robust conclusions about the effects of performance level on engagement patterns and could test targeted interventions to support lower-performing students’ AI interaction capabilities. Additionally, combining case study depth with quasi-experimental comparison would achieve both contextual understanding and stronger generalizability across diverse educational settings.

Third, the single-session design, while enabling controlled observation of immediate interaction patterns, cannot capture the evolutionary nature of human-AI relationships in design education. Students’ engagement strategies likely develop sophistication over time through repeated exposure and practice. Longitudinal studies tracking students’ ChatGPT engagement across multiple design projects over a semester or year-long periods would provide crucial insights into skill development trajectories and long-term adaptation patterns.

Fourth, the absence of final design output analysis represents a significant methodological constraint. While systematic observation of revision processes provides evidence of differential engagement strategies, we cannot definitively establish whether these behavioral and cognitive differences translated into superior design quality. This limitation reflects a deliberate methodological choice to focus on psychological processes rather than outcomes, but it constrains our ability to make claims about effectiveness. Future research should integrate design output assessment using expert evaluation or standardized rubrics alongside process analysis, enabling investigation of the process-outcome relationship.

Fifth, this study focused exclusively on individual feedback processing in self-directed learning modes, without addressing the roles of peer collaboration and teacher guidance in AI-mediated design education environments. The integration of ChatGPT feedback with traditional collaborative design processes remains unexplored. Future research could introduce collaborative learning frameworks to investigate how students negotiate between AI feedback, peer input, and instructor guidance, and how these multiple feedback sources interact to influence design outcomes. In addition, the current study relied primarily on behavioral observation and self-reported measures to assess engagement patterns. Recent research has demonstrated the value of physiological measures in understanding human-computer interaction in creative contexts. Recent studies have shown that EEG feedback can provide objective indicators of cognitive load and creativity performance, while haptic feedback systems can enhance creative engagement (Wu et al., 2025; Wu and Wang, 2025). Future research could benefit from incorporating such physiological measures to provide more objective and real-time indicators of the psychological processes underlying student engagement with AI feedback systems, potentially revealing cognitive and emotional dynamics not captured through behavioral coding and self-reports alone.

## Data Availability

The original contributions presented in the study are included in the article/Supplementary material, further inquiries can be directed to the corresponding authors.
