# Supplementary material for: Psychological engagement with automated design improvement feedback: a multiple case study of ChatGPT in design education
Source: Front Psychol. 2026 Feb 16;17:1729241. doi: 10.3389/fpsyg.2026.1729241 (PMC12950771; doi:10.3389/fpsyg.2026.1729241)
Supplement: Supplementary file 1 [file Supplementary_file_1.docx]

Supplementary Material

**Appendix A: Reflection log**

| Number: | | Date: |
| --- | --- | --- |
| **ChatGPT interaction log** | | |
| Number of prompts written |  | |
| Types of prompts used | □basic prompt □provide specific design specifications □provide target user information □request feedback in specific style and tone □request solution validation □supplement additional specific requirements □request to narrow down the feedback focus range □provide reference cases □express satisfaction/dissatisfaction with feedback □request feedback improvement □completely regenerate feedback  □Other：_________ | |
| Most effective prompt |  | |
| Least effective prompt |  | |
| **Feedback processing** | | |
| Number of feedback points | |  |
| Accepted feedback | | Quantity: _____ Example: |
| Rejected feedback | | Quantity: _____ Reason: |
| Modified feedback | | Quantity: _____ How it was modified: |
| **Reflection points** | | |
| Most valuable feedback this week | | |
| Challenges encountered | | |
| Areas for design improvement | | |
| Willingness to continue using ChatGPT | | |

**Appendix B: Design task worksheet**

| Number: | | Date: | |
| --- | --- | --- | --- |
| Tesign task theme： | **Smart Chair Design**  Please select a specific group of people (e.g., the elderly, children, long-term sedentary office workers, individuals with special needs, etc.) and design a smart chair for them. Analyse the target users’ needs and pain points, come up with strategies to address them, and provide a detailed description of the product, including the target group, design objectives, design explanation, structure and functions, smart interaction methods, technical feasibility, and competitive market advantages. (Word count: 500-1000 words). | | |
| **Initial design proposal description：** | | | |
| Must include the following key points：  □ design background and objectives□ needs analysis□ solution□ implementation steps□ expected outcomes□ risk assessment | | | |
| **ChatGPT feedback process** | | | |
| First round of feedback | | Second round of feedback | |
| Prompt design (select focus points):□ solution logic analysis□ feasibility evaluation□ completeness check□ specific implementation  suggestions□ risk identification | | In-depth feedback focus:□ detailed refinement□ solution optimisation suggestions□ additional explanations of  requirements□ other：_______ | |
| Feedback received: | | Supplementary feedback: | |
| Planned modifications: | 1. | Additional modifications: | 1. |
|  | 2. |  | 2. |
|  | 3. |  | 3. |
|  | 4. |  | 4. |
|  | 5. |  | 5. |
| **Revision process** | | | |
| Revision record:  Type of revision:□ logic optimisation□ feasibility enhancement□ content supplementation□ process adjustment  □ risk response□ other: ___________ | | | |
| Reason for revision: | | | |
| **Final solution** | | | |
| **Content of the optimised solution:**  Summary of major improvement points:1.2.3. | | | |
| **Process evaluation and reflection** | | | |
| Time investment：  Plan writing: ___ minutesFeedback received: ___ minutesPlan revision: ___ minutesTotal time: ___ minutes | | Most valuable feedback suggestions:  Reasons for not adopting certain suggestions:  Main takeaways from this revision:  Record of remaining issues: | |
| **Review evaluation** | | | |
| Review comments | | Plan completeness: ○ Low ○Medium ○HighPlan feasibility: ○ Low ○Medium ○HighRevision effectiveness: ○ Low ○Medium ○High  Further recommendations: | |

**Appendix C: Interview coding scheme**

| **Primary code** | **Code** | **Secondary code** | **Description** |
| --- | --- | --- | --- |
| Emotional response  (ER) | ER-P | Positive emotion | Express positive emotional experiences |
|  | ER-N | Negative emotion | Express negative emotional experiences |
|  | ER-M | Mixed emotion | Express complex or contradictory emotions |
| Design experience  (DE) | DE-P | Positive experience | Express satisfaction and gains in design |
|  | DE-N | Negative experience | Express dissatisfaction and difficulties |
|  | DE-M | Neutral description | Objective description of design process |
| Challenges & needs  (CN) | CN-T | Technical challenges | Describe technical difficulties in usage |
|  | CN-C | Cognitive load | Describe cognitive pressure in design |
|  | CN-TI | Time Cost | Describe time investment issues |
|  | CN-S | Support needs | Express needs for additional support |
| Design strategy application (LS) | DS-A | Active adjustment | Describe active improvement of design methods |
|  | DS-P | Passive acceptance | Describe passive acceptance of feedback |
|  | DS-S | Selective adoption | Describe selective adoption of feedback |
| Future usage intention  (FI) | FI-C | Continue usage | Express willingness to continue using |
|  | FI-CO | Conditional usage | Express conditional willingness to continue |
|  | FI-S | Stop usage | Express intention to stop using |

***Interview questions***

|  | **Interview questions** |
| --- | --- |
| 1 | How would you describe your overall experience using ChatGPT for design feedback? |
| 2 | What changes have you noticed in your design process during today’s session? |
| 3 | How do you typically request design feedback from ChatGPT? |
| 4 | What specific strategies do you use to get more effective feedback? |
| 5 | How do you usually process the feedback you receive from ChatGPT? |
| 6 | How much of ChatGPT’s suggested revisions do you adopt? Why? |
| 7 | How do you evaluate the quality of feedback provided by ChatGPT? |
| 8 | What considerations do you take into account when processing feedback? |
| 9 | How do you decide whether to accept or reject specific feedback suggestions? |
| 10 | How do you integrate the feedback into your design modifications? |
| 11 | How do you feel about getting feedback from ChatGPT? |
| 12 | Compared to traditional teacher feedback, what different feelings do you have about AI feedback? |
| 13 | How much trust do you have in the feedback provided by ChatGPT? |
| 14 | Do you plan to continue using ChatGPT for design feedback in the future? Why? |
| 15 | What difficulties have you encountered when using ChatGPT for feedback? |
| 16 | What improvements are most needed if ChatGPT is to be used on a larger scale? |

***Interview questions and codes mapping***

| **Interview question number** | **Primary corresponding codes** | **Possible secondary codes** |
| --- | --- | --- |
| Q1-2 (General emotional experience) | ER-P, ER-N | ER-M, DE-P, DE-N |
| Q3-4 (Emotional needs in feedback) | DS-A, ER-M | CN-C, DS-S |
| Q5-6 (Emotional responses to revisions) | DS-S, ER-M | DE-P, DE-N |
| Q7-8 (Emotional self-awareness) | ER-M, CN-C | DS-A |
| Q9-10 (Emotional coping strategies) | DS-S, ER-M | DS-A, DE-M |
| Q11-12 (Comparative emotional responses) | ER-P, ER-N | ER-M |
| Q13-14 (Emotional trust & commitment) | ER-M, FI-C | FI-CO, CN-S |
| Q15-16 (Emotional challenges & support needs) | CN-T, ER-N | CN-S, CN-C |

Coding Instructions:

1. Each interview question has its primary corresponding code categories
2. Respondents’ answers may involve multiple code categories
3. Coders need to judge which codes to use based on specific context
4. A single text segment can be assigned multiple code labels
5. Regular checks of coding consistency and accuracy are required

**Appendix D: Cognitive engagement coding**

| **Code** | **Coding definition** | **Judgment criteria** | **Exclusions** | **Examples** |
| --- | --- | --- | --- | --- |
| P | Purpose statement：  clearly stating the objectives or direction for this design | - Must include “want/need/plan” or similar goal-oriented words and specify concrete design objectives | - Casual conversations without specific design objectives | - “I want to improve the navigation design layout”. - “I need to redesign this interface to be more intuitive”. |
| Q | New design query：  posing new specific design-related questions to ChatGPT | - First sentence must contain specific design solution inquiries | - Requesting repetition or modification of existing suggestions | - “What would make this design more engaging”? - “What kind of UI elements should be included here”? |
| R | Feedback processing：  participant reading and reviewing ChatGPT’s responses | - Clear reading comprehension behaviour - Deliberate review of specific content | - Quick scanning without meaningful engagement | - Participant carefully reads Claude’s response, actively engages with the response content |
| M | Modification interaction：  requesting modifications or supplements to ChatGPT’s suggestions | • Requesting elaboration/clarification of given suggestions   - Requesting supplements or refinements to existing suggestions | - New queries unrelated to previous suggestions | - “Could you elaborate on this part”? - “Could this suggestion be more detailed”? |
| D | Design decision：  clear decisions made regarding ChatGPT’s suggestions | - Clear expression of acceptance or rejection - Clear explanation of partial acceptance with reasoning | - Ambiguous responses like “Let me think about it” | - “This suggestion is good, I’ll adopt it” - “This part doesn’t fit, I’ll skip it” |
| I | Implementation decision Making：  incorporating confirmed modifications into the design | - Actions of recording or modifying design solutions | - Just showing or discussing modifications without implementation plans | - Participant records or directly modifies design solutions |
